# Late onset adrenal insufficiency after adrenalectomy due to latent nonclassical 21-hydroxylase deficiency: A case report

**DOI:** 10.1097/MD.0000000000011888

**Published:** 2018-08-17

**Authors:** Hiroyuki Hirai, Keisuke Kuwana, Yoshiro Kusano

**Affiliations:** a Department of Internal Medicine, Shirakawa Kosei General Hospital, Shirakawa City; b Department of Diabetes, Endocrinology and Metabolism School of Medicine, Fukushima Medical University, Fukushima City, Fukushima, Japan.

**Keywords:** 21-hydroxylase deficiency, adrenal incidentaloma, adrenal insufficiency, adrenalectomy, nonclassical 21-hydroxylase deficiency

## Abstract

**Rationale:**

Adrenal incidentaloma is sometimes complicated with 21-hydroxylase deficiency (21-OHD). Latent nonclassical 21-OHD in incidentaloma is difficult to diagnose. Although adrenalectomy in 21-OHD has been conducted when malignancy could not be excluded, adrenal insufficiency sometimes occurs, and it might not be observed immediately after operation. Here, we report a case of a 71-year-old man who experienced adrenal insufficiency over 2 decades postadrenalectomy, leading to a diagnosis of latent nonclassical 21-OHD.

**Patient concerns:**

A 71-year-old man was admitted to the hospital due to difficulty in movements and a sodium level of 119 mEq/L. His medical history revealed precocious puberty and left adrenalectomy because of an incidentaloma at 49 years of age, diagnosed pathologically as an adenoma. He did not attend follow-up visits because he did not have any symptoms. In 2017, 3 months before hospitalization, he experienced general fatigue. A few days before admittance, he complained of difficulty in moving and visual hallucination of small animals.

**Diagnoses:**

Laboratory evaluations revealed a high level of adrenocorticotropic hormone (ACTH) and low cortisol level. ACTH-stimulating test revealed a low basal level and low response for cortisol, and a high basal level and low response for 17-hydroxyprogesterone. We analyzed large gene deletion or conversion and the 9 most common micro mutations in the *CYP21A2* gene by polymerase chain reaction; micro mutation of I172N and heterozygous large gene deletion or conversion were detected leading to the diagnosis of nonclassical 21-OHD.

**Interventions:**

Immediately, 100 mg hydrocortisone was administered, followed by daily hydrocortisone and saline. The symptoms and hyponatremia improved in a few days. He was discharged from the hospital on day 34 with a daily dose of 15 mg hydrocortisone.

**Lessons:**

Clinicians should be aware of late onset of adrenal insufficiency after adrenalectomy. In such cases, clinicians should not overlook the latent nonclassical 21-OHD.

## 1 Introduction

21-Hydroxylase deficiency (21-OHD) is an autosomal recessive disease resulting from mutations in the *CYP21A2* gene. Depending on the residual 21-hydroxylase activity, clinical phenotypes consist of salt wasting, simple virilizing, and nonclassical.^[1,2]^ The prevalence of classical 21-OHD has been reported as approximately one in 15,000 live births^[1]^; the nonclassical prevalence varies depending on race.^[1,3]^ Kashimada et al reported the prevalence of nonclassical 21-OHD to be about 0.58 per 1,000,000 in Japan.^[4]^

Classical or nonclassical 21-OHD has been reported to complicate adrenal incidentaloma.^[1,2]^ However, the diagnosis of latent complicated nonclassical 21-OHD in incidentaloma is sometimes difficult because of few symptoms.^[1,2]^ In addition, adrenalectomy is not performed on the background of 21-OHD because of the risk of future adrenal crisis, unless malignancy could not be excluded when adrenalectomy is occasionally performed.^[5,6]^ Although adrenal insufficiency usually occurs immediately after adrenalectomy,^[7]^ this is not always the case. Therefore, the strategy of handling adrenal incidentaloma in patients with 21-OHD still remains to be unified.

Here, we report a rare case of adrenal insufficiency appearing over 2 decades after adrenalectomy leading to the diagnosis of nonclassical 21-OHD.

## 2 Case presentation

In October 2017, a 71-year-old man visited our hospital due to moving difficulties and visual hallucination after experiencing general fatigue for 3 months. He reported dizziness and increased fatigue, 3 weeks prior to presentation, followed by urinary frequency, urinary incontinence, and a fall, 2 weeks later. To investigate his symptoms, head computed tomography (CT) and blood examination were conducted, which revealed a sodium level of 119 mEq/L, and he was hospitalized. Upon admittance, the patient underwent a physical examination that revealed a height of 138.5 cm, a weight of 49.0 kg, and a body mass index of 25.5 kg/m^2^. His temperature was 36.2°C and blood pressure 127/67 mm Hg. Pigmentations were detected, which were more visible around the lips, on the tongue, and fingers. Although the penis and scrotum were normal in size, the testicles measured small on an ultrasound scan.

His medical history revealed a growth spurt at approximately 8 years of age and the appearance of pubic hair at 10. His height did not increase past 139 cm from the age of 12. In his thirties, he suspected infertility, which was not investigated. At 49 years old in January 1996, he underwent a left adrenalectomy because of a heterogenous incidentaloma, with a size of approximately 3 to 4 cm. Laboratory investigations at the time revealed adrenocorticotropic hormone (ACTH): 102.2 (7.2–63.3) pg/mL, cortisol: 14.6 (6.24–18.0) μg/dL. 17-Ketosteroids (17-KS) were also reported to be elevated. The detailed results of these tests are summarized in Table 1. Because malignancy could not be fully excluded, adrenalectomy was performed. The mass was pathologically diagnosed as an adenoma and not malignant. After operation, although he was prescribed prednisolone, he did not keep up with his follow-up visits at the hospital because of no symptoms.

**Table 1:**
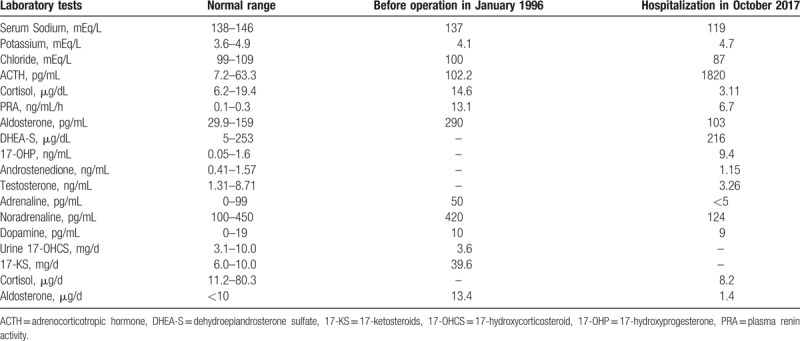
Comparison of laboratory results prior to adrenalectomy and at the most recent hospitalization.

**Figure 1:**
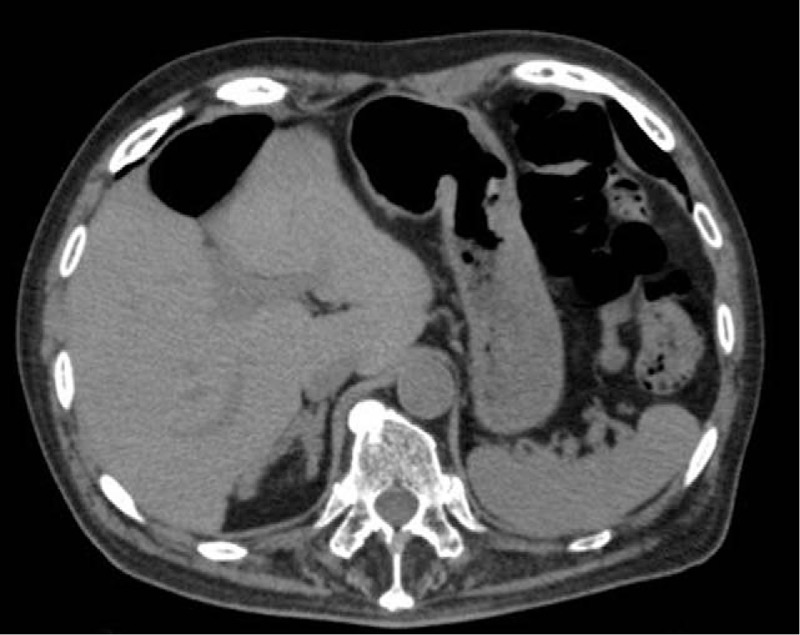
Plain abdominal computed tomography shows an almost normal-sized right adrenal gland and no residual left adrenal gland.

Abdominal CT revealed almost normal right adrenal gland (Fig. 1). Laboratory evaluations revealed ACTH level of 1820 (7.2–63.3) pg/mL and cortisol level of 3.11 (6.24–18.0) μg/dL. The results of the other tests are presented in Table 1. Therefore, a diagnosis of primary adrenal insufficiency was suspected, and the patient immediately received a dose of 100 mg hydrocortisone intravenously. This initial dose was followed by a maintenance regimen of intravenous hydrocortisone diluted in saline as follows: 200 mg for 2 days, 150 mg for 3 days, 100 mg for 3 days, and 50 mg for 3 days. Thereafter, he received 15 mg hydrocortisone orally. During this time, the patient also received approximately a liter of physiologic saline per day. Sodium levels were monitored and reached 126 meq/L on day 2, 131 meq/L on day 3, and 138 meq/L on day 7 after hospitalization. As a result, the patient's symptoms improved after a few days. An ACTH-stimulating test was performed, with the results revealing low response of cortisol 17-hydroxyprogesterone (17-OHP). The basal levels were low in the former and high in the latter. The complete results are summarized in Table 2. Therefore, a diagnosis of primary adrenal insufficiency was made. No anti-adrenal cortex antibody was detected. The patient was discharged on day 34 with a daily oral dose of 15 mg of hydrocortisone.

**Table 2:**
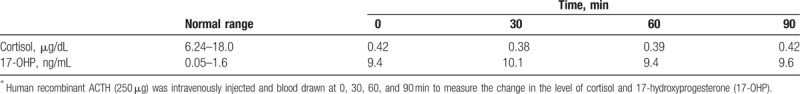
Results of the adrenocorticotropic hormone (ACTH)-stimulating test^∗^.

We suspected nonclassical 21-OHD, and searched for the most common *CYP21A2* mutations by polymerase chain reaction (PCR) as per previous studies,^[8,9]^ using the primers previously reported for these mutations,^[9]^ after obtaining the patient's informed consent, looking for the large gene deletion or conversion, and the following 9 micro conversion-derived mutations: P30L in exon 1, 656A/C > G in intron 2, I172N in exon 4, V281L in exon 7, Q318X in exon 8, R356W in exon 8, the cluster I236N, V237E and M239K in exon 6, L307+T in exon 7, and 8 bp-del in exon 3.^[8,9]^ The mutations positive in our patient were micro mutation I172N and heterozygous large gene deletion or conversion leading to the diagnosis of nonclassical 21-OHD.

## 3 Discussion

The patient in our study presents 2 concerns: first, adrenal incidentaloma is occasionally complicated with nonclassical 21-OHD, and second, adrenal insufficiency in nonclassical 21-OHD can occur as late as 2 decades after adrenalectomy, issues that can be overlooked by clinicians.

Patócs et al reported that the prevalence of germline CYP21 mutations in patients with bilateral and unilateral adrenal incidentaloma were 21.1% and 16.1%, respectively^[10]^; however, accurate prevalence of adrenal incidentaloma with nonclassical 21-OHD still remains unclear. Although menstrual irregularity due to overproduction of androgen leads to diagnosis in women, there are often no symptoms in nonclassical cases of 21-OHD in men making the diagnosis difficult^[1]^; therefore, the evaluation of 17-OHP is important. In the case of our patient, although premature puberty was present 17-OHP was not measured before adrenalectomy. The high value of 17-KS does not contradict the excess of androgen secretion. The level of 17-OHP in 2017 did not noticeably change after ACTH stimulation. Ferreira et al had previously reported a similar finding and had reasoned that since the adrenal cells were already maximally stimulated by chronically elevated ACTH, the value of basal 17-OHP was already high and residual rising capacity might be absent, leading to a minute change in its levels following external ACTH stimulation.^[11]^

Recently, Hanna et al reported that if adrenal incidentalomas are lipid rich, do not overproduce hormones, and are <4 cm in diameter, no further investigation is required.^[12]^ However, no guidelines in dealing with adrenal incidentalomas were in place in the 1990s. Cases of adrenal incidentaloma in 21-OHD has mostly been reported as normal or myelolipoma.^[1,13]^ However, since cases of adenocarcinoma in nonclassical 21-OHD has also been reported,^[6]^ clinicians should consider management of adrenal incidentalomas in these patients with care, and there is a need for appropriate criteria for adrenalectomy in 21-OHD. In the case of our patient, the tumor was heterogeneous, 3 to 4 cm size, and the level of 17-KS was high, making the exclusion of malignancy difficult. The latent appearance of adrenal insufficiency in this patient, 2 decades after adrenalectomy, and despite the undiagnosed nonclassical 21-OHD might be due to a sustained function of the residual adrenal gland.

In general, if one adrenal gland remains after adrenalectomy, chronic adrenal insufficiency may not easily occur.^[14]^ However, Yoshiji et al recently reported a case of chronic adrenal insufficiency after adrenalectomy due to renal cell carcinoma.^[15]^ In addition, Mitchell et al reported that approximately 20% of patients without hyper-cortisol secretion will experience adrenal insufficiency after unilateral adrenalectomy.^[16]^ In both these studies, the onset of adrenal insufficiency was often immediately after adrenalectomy.^[15,16]^ Nagasaka et al reported a case of silent 21-OHD with acute onset persistent adrenal insufficiency after unilateral adrenalectomy.^[7]^ Therefore, clinicians should be wary of the acute onset of adrenal insufficiency postadrenalectomy. The fact that our patient presented with a late-onset adrenal insufficiency makes this a rare case, and to the best of our knowledge, this is the first report of such a delayed occurrence of insufficiency after unilateral adrenalectomy in nonclassical 21-OHD. Such a late onset adrenal insufficiency in this case could be because the other adrenal gland was functioning relatively well, as proved by adosterol scintigraphy.^[7]^ No major complications such as pulmonary embolism or sepsis occurred during the operation. Besides, the *CYP21A2* mutation pattern might allow for some residual 21-hydroxylase activity.

Our study had a few limitations: first, in this case, the mutations of I172N and heterozygous large gene deletion or conversion are not consistent with the reported P30L mutation or compound heterogeneous type often reported in nonclassical 21-OHD in Japan.^[2,4]^ I172N is often detected in simple virilizing or nonclassical 21-OHD, and has been reported to retain some residual 21 hydroxylase activity, causing virilization signs or precocious puberty through androgen overproduction.^[1,2,8,9]^ Unfortunately, we could not evaluate the occurrence of these mutations in his family, as his parents were already dead and his brothers do not live close by. In addition, we only tested for major mutations previously reported,^[8,9]^ leading to inadequate analysis of the *CYP21A2* gene and other genetic forms such as homozygous and heterozygous expression. Second, the values reported for hormones in 2017 were different from that of 1996 (Table 2), which might be due to the influence of 21-OHD, the removal of only one adrenal gland, and aging. To assess this hypothesis, we needed to perform further tests on the residual specimen of the removed adrenal gland. Unfortunately, we could not access neither imaging reports nor pathologic specimens of the removed adrenal tumor, making further evaluations impossible.

## 4 Conclusion

In conclusion, clinicians should be wary of late onset adrenal insufficiency after adrenalectomy, and not overlook latent nonclassical 21-OHD.

## Acknowledgments

The authors thank LSI Medience Corporation, Tokyo, Japan for performing PCR tests. The authors also thank Editage (www.editage.jp) for English language editing.

## Author contributions

**Investigation:** Keisuke Kuwana.

**Writing – original draft:** Hiroyuki Hirai.

**Writing – review & editing:** Yoshiro Kusano.
